# The Sanative Influence of Climate

**Published:** 1846-10-01

**Authors:** 


### THE SANATIVE INFLUENCE OF CLIMATE. By Sir *James Clark*, Bart., M.D. F.R.S. Fourth Edition, royal 12mo. pp. 412. London, 1846.

A WORK of long-established reputation like the present seldom calls for more than a passing notice in a quarterly review, unless, indeed, the quantity of additional matter which a new edition contains be such as fairly to demand a more ample examination. That the fourth edition of the "Sanative Influence of Climate" has been carefully revised by its distinguished author, and that its contents have been considerably enlarged, is quite true; but it is not so much for this reason that we are induced to devote a page or two to its consideration, as for the purpose of expressing our earnest caution to young medical men against attaching so great an importance in the treatment of many maladies to a change of climate from our own shores to distant countries as has been, and we fear still is, too frequently done. That a vast deal of suffering and positive mischief has often been committed by physicians indiscreetly sending patients affected with consumption, to different parts of the continent and other regions, in the vain hope of a southern climate being capable of effecting what no spot in our own country was supposed able to do, cannot be disputed by any one who is at all acquainted with the melancholy history of that disease. Some of the very places, which, a few years ago, were so strongly recommended as most adviseable residences for phthisical invalids, are now admitted to be utterly inappropriate. Nice, for example, once stood very high in estimation ; and now what does our experienced author tell us ?—" little benefit is to be expected from the climate....... Indeed the cases of consumption which ought to be sent to this place are of rare occurrence." And what do we learn from Drs. Renton and Heineken respecting the majority of the poor sufferers that are sent out to Madeira ? " Of the 35 cases reported by Dr. H., several died before they reached the island, three within a month of their landing, and five or six in about six months. Of 47 cases of the same class of invalids in Dr. R.'s report, more than two-thirds died within six months of their arrival in the island." Was it not a cruel and disgraceful thing on the part of medical men to have lent their sanction to, nay, to have suggested the propriety of, a distant change of climate in the greater number of these melancholy cases ? Without pursuing this subject further, we have no hesitation in expressing our decided opinion that the favourite practice of sending consumptive patients to Madeira and various places in the South of Europe has, within the last 40 years, been productive of much greater harm than good. Let it never be forgotten that it is almost solely in the threatened and incipient cases that any decided benefit can be anticipated from a residence in a warm climate. That the young members, more especially the sons, of a consumptive family may often have their health and strength improved by residing for a few years in Madeira or one of the West India Islands, we are ready to admit; but, if the tubercular disease be fairly established, we are only precipitating the mischief by advising such a change.

In the treatment of the different forms of Dyspepsia, Sir James Clark is inclined to lay much more stress upon the particular locality abroad to be recommended than we should deem at all necessary. That change of air, change of scene, change of diet, and change of occupation are better remedies in a host of dyspeptic and hypochondriacal affections than all the pharmaceutical formulæ in the world, who will deny ? But surely all these things might be generally had within the limits of our own dear land, if people were but brought to think so.

We fear that Sir James's book has had the effect of making many, especially among the wealthier classes, imagine that they must forthwith go upon the Continent to find relief for certain stomach ailments, which might be just as readily got rid of in their own country, provided some very simple hygienic rules were attended to. The following passage will serve as an example of what we think may sometimes be fairly objected to the present work ; viz. in attaching an undue importance to mere peculiarity of climate in the treatment of different morbid affections. After describing the different forms of Dyspepsia, Sir James tells us that each form requires a different climate for its relief:

"The patient with gastritic dyspepsia should not, for example, go to Nice, nor the South-east of France. In such cases, the South-west of France or Devonshire is preferable, and Rome and Pisa are the best places in Italy. On the other hand, in atonic dyspepsia, in which languor and sluggishness of the system, as well as of the digestive organs, prevail, with lowness of spirits and hypochondriasis, Nice is to be preferred to all the other places mentioned; and Naples will generally agree better than Rome or Pisa; while the South-west of France and Devonshire, and all similar climates, will be injurious. In the nervous form of dyspepsia, a climate of a medium character is the best, and the choice should be regulated according as there is a disposition to the gastritic or the atonic form."

Now in truth Dyspepsia, with all its accompanying and induced disorders, is generally much more influenced by the food that is taken into the stomach than by the air that is inhaled into the lungs. Travelling about, too, from one place to another is, on the whole, vastly preferable in all such cases to a stationary residence in any spot, however genial may be its climate. If people *must* go abroad, then it may be perfectly true that "Rome is the best residence in Italy in gastritic dyspepsia, and Nice the best climate in the purer cases of atonic dyspepsia;" but we verily believe that the soft air of Devonshire or the Isle of Wight, and the keener breezes of Wales and Scotland would answer quite as well for the health, and a great deal better for the purse, and often for the morals too.

Within the last five years there has started into notice a new candidate for hygienic distinction as a resort for invalids;—Egypt. Sir James, in the present edition of his work has, for the first time, drawn the attention of his readers to the land of old Nile.

We give the following extract as containing his opinion, formed not from personal examination, be it remembered, but from what has been published by Clot-Bey, Dr. Cumming, and one or two other writers.

"From the description which has been given of the climate, may be inferred the character of the diseases and deranged states of health which will derive benefit from a Winter passed in Egypt. Invalids requiring a dry, warm, exhilarating climate will find it here. Certain forms of dyspeptic disorder of long standing, with their consequences, chronic affections of the mucous membranes of the respiratory and other organs of an atonic and congestive kind, and chronic rheumatism may be numbered as among the diseases likely to be benefited; and there is a large class of persons suffering from a state of deranged health which scarcely admits of definition, and yet is well known as one of the many consequences of sedentary habits, prolonged and anxious mental exertions, irregularity of living, &c. who will derive great advantage from a Winter spent on the Nile. But the patients, whom I have had chiefly in view in giving this brief notice of the climate of Egypt, are young men whose health has become deranged from the causes already mentioned, or from others of an analogous kind, and to such a degree as to excite apprehension lest the disordered condition of their system might end in consumption. For this class of invalids, suffering from disordered health rather than actual disease, and not so delicate as to be injured by the inconveniences and mode of life to which they would be subjected during a Winter in Upper Egypt, the climate seems to me peculiarly well adapted. In the case of invalids also, returning from India for the recovery of their health, and to whom it is important to avoid arriving in England at an unfavorable season, it may be very advantageous to remain in Egypt till the most favorable period of the year arrives for their return home."

We need scarcely say that there are many regions of the earth where that best of blessings, health, is much more likely to be found than in Egypt.

If our space had permitted, we should like to have extracted some of Sir James' excellent remarks on the importance of proper ventilation of rooms, in the treatment of many maladies. This is a point that has hitherto been far too little attended to; and it is to be much desired that our author's sound advice will not be lost upon the public.

In conclusion, we have again the pleasure of recommending Sir James' work as *the* guide-book to direct medical men in their selection of the proper climate, for those cases where a change of residence may be deemed adviseable.

